# Diet of the U. S. Army Soldier in the Training Camp

**Published:** 1919-01

**Authors:** 


***Diet of the U. S. Army Soldier in the Training Camp.*** By J. R. Murlin, Major S. C., N. A. *Journal of the American Medical Association.* Sept. 21, 1918.

The Food Division of the Surgeon General's Office having completed surveys of 227 messes distributed among 40 training camps in America (about 60,000 men in all), the author proposes to study and discuss the results obtained, after briefly describing the messing system of the U. S. Army.

Soldiers are fed by companies or units corresponding in size. The company commander is responsible for the proper feeding of the troops, and a subaltern, known as mess officer, supervises the mess. Purchases are made by the mess sergeant. Fresh meat and bread are issued daily by the camp quartermaster, while other articles of food are distributed every 10 days on requisition by the mess sergeant. The latter may in some cases obtain money instead of the goods and purchase the undrawn portion of the ration at local markets. This portion is on an average 35 o/o of the total ration and must not exceed 50 o/o.

Table 1 gives the garrison ration. The average value of the ration, according to latest estimates, is 39 cents per man, per day.

The object of the surveys was to estimate the quantity of food supplied, wasted, and consumed, and to give instructions to the mess officers and sergeants as to the proper use of same. An inventory of the stock on hand was made at the beginning and the close of a given period, the edible waste was weighed, mixed, sampled and analyzed. Deductions were then made for wastage, thus giving the actual consumption in grams of protein, fat, and carbohydrate per man per day.

A table follows giving a summary of nutritional surveys, and showing the average amount of nutrients supplied, wasted, and consumed for 49, 68, 85, 143, 185 and 213 messes. The total amount of energy supplied is a little under 4,000 calories, the waste about 300 calories and the amount consumed about 3,700 calories. The distribution of calories consumed is 14 o/o protein, 31 o/o fat, and 55 o/o carbohydrate with a small improvement in the amount of waste. The actual cost of the ration consumed at present is 42.75 cents, which is therefore higher than the value of the ration. This is due to the fact that, owing to the absence of a certain number of men from Saturday to Sunday night, the attendance at mess is only about 90 o/o of the total number of rations authorized.

Another table follows showing the average consumption of food.

The author notes that the army rations, as laid down by army regulations, had to be modified according to requirements. The original army ration, besides being excessive, would provide too much acid ash and protein. As a consequence of this, the author wonders whether it would not be advisable to readjust the army rations to a more adequate basis. The advantages of such modification would be as follows : (1) reduction of waste; (2) more satisfactory distribution of nutrients; (3) centralization of purchasing, etc. A distinction should, however, be made between "training" and "field" rations as is the case in the French, British, and Italian armies. The present garrison ration of the U. S. army is about 500 calories in excess of all army rations used by the Allied forces.

The author insists upon the necessity of treating the messing of troops in training camps as a hygienic measure and shows the importance of physical fitness of the specialized soldier, airman, artillery observer, tank operator, in modern warfare. He then gives a short account of the work already carried out by the Food Division, and states that the question of food economy has been much improved lately in the training camps, but that a number of problems in connection with the feeding of troops are still pending to which he calls the attention of medical men.

